# Supplementary material for: The Fungus Beauveria bassiana Alters Amounts of Sterols, Fatty Acids, and Hydroxycinnamic Acids in Potato Solanum tuberosum
Source: Plants (Basel). 2023 Nov 22;12(23):3938. doi: 10.3390/plants12233938 (PMC10707819; doi:10.3390/plants12233938)
Supplement: Supplementary file 1 [file plants-12-03938-s001.zip › plants-2655135-Supplementary Materials.pdf]

Supplementary Materials

The Fungus *Beauveria bassiana* Alters Amounts of Sterols, Fatty Acids, and Hydroxycinnamic Acids in Potato *Solanum tuberosum*

Maksim Tyurin [1], Elena Chernyak [2], Oksana Tomilova [1,3], Khristina Tolokonnikova [1], Svetlana M. Malysh [3],
Elena Khramova [4], Sergey Morozov [2] and Vadim Kryukov [1,*]

[1] Institute of Systematics and Ecology of Animals, Siberian Branch of Russian Academy of Sciences (SB RAS), Frunze Str. 11, Novosibirsk 630091, Russia; maktolt@mail.ru (M.T.); toksina@mail.ru (O.T.); klatty@yandex.ru (K.T.)

[2] N.N. Vorozhtsov Novosibirsk Institute of Organic Chemistry, SB RAS, Academician Lavrentyev Ave. 9, Novosibirsk 630090, Russia; chernyak@nioch.nsc.ru (E.C.); morozov@nioch.nsc.ru (S.M.)

[3] All-Russian Institute of Plant Protection, Podbel'skogo Str. 3, St. Petersburg 196608, Russia; s.malysh-vizr@yandex.ru

[4] Central Siberian Botanical Garden SB RAS, Zolotodolinskaya Str. 101, Novosibirsk 630090, Russia; elenakhramova2023@yandex.ru

* Correspondence: krukoff@mail.ru

Table S1. Origin and identity of the secreted-lipase sequence from *Beauveria bassiana* strains and Sar-31 reisolates used in the present study.

Strain name (insect host order, family)	Sampling site coordinates and year	Nucleotide sequence identity, %							
		SAR-31	SAR-31 reisolate 1	SAR-31 reisolate 2	SAR-31 reisolate 3	BLDR-09	BBK-1	Bca2(m)-09	GusB3
SAR-31 (Orthoptera, Acrididae)	53°41′N 78°02′E (2001)	ID				-	-	-	-
SAR-31 reisolate 1	-	100	ID						
SAR-31 reisolate 2	-	100	100	ID					
SAR-31 reisolate 3	-	100	100	100	ID				
BLDR-09 (Coleoptera, Chrysomelidae)	47°14′N 39°42′E (2009)	87.8	87.8	87.8	87.8	ID	-	-	-
Bos-13 (Lepidoptera, Crambidae)	45°12′N 40°47′E (2013)	99.7	99.7	99.7	99.7	88	ID	-	-
BBK-1 (Orthoptera, Acrididae)	53°44′N 78°02′E (2000)	88	88	88	88	99.2	88.3	ID	-
Bca2(m)-09 (Coleoptera, Carabidae)	40°54′N 68°29′E (2009)	91	91	91	91	95.3	91.2	95	ID
GusB3 (Lepidoptera, Crambidae)	51°50′N 108°15′E (2009)	95.7	95.7	95.7	95.7	89.5	95.9	89.5	91.1

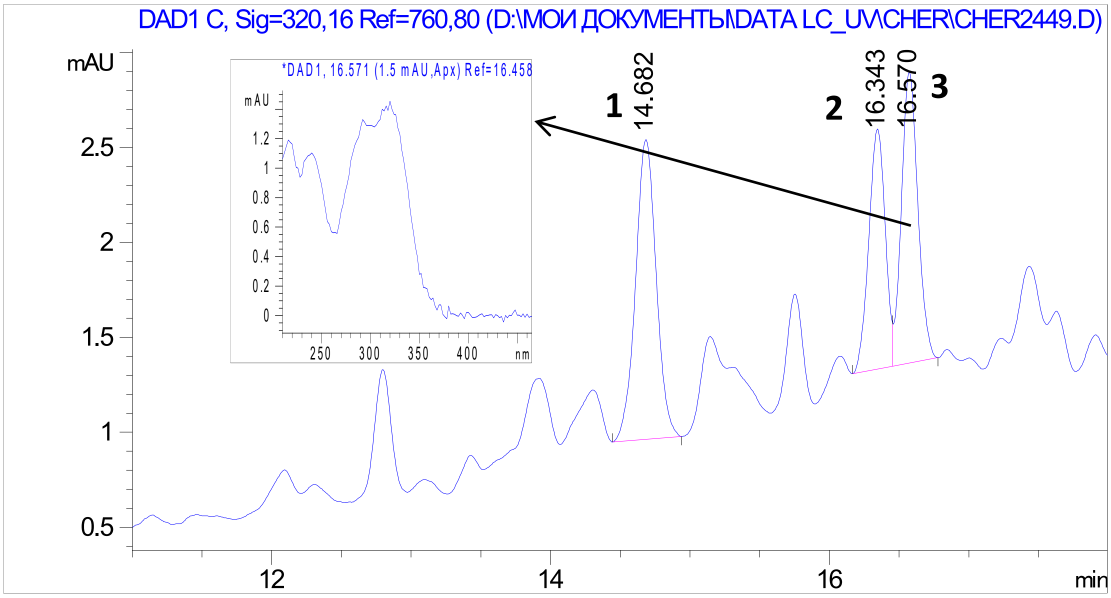

Figure S1. The chromatographic profile of hydroxycinnamic acids in a potato root sample (λ = 320 nm). Peak 1: chlorogenic acid, peak 2: an unidentified hydroxycinnamic acid, peak 3: caffeic acid. Chlorogenic acid (Sigma-Aldrich) was used for quantification of hydroxycinnamic acids. Identification of other acids was carried out on the basis of UV spectra and retention times of the peaks (Chamorro et al., 2021).

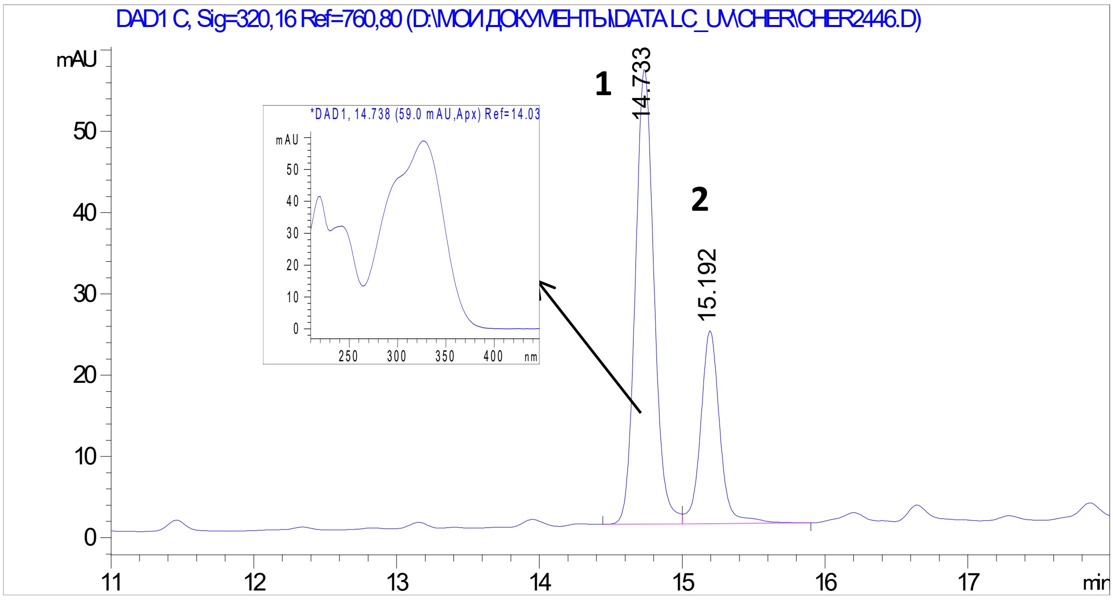

Figure S2. The chromatographic profile of hydroxycinnamic acids from potato leaves (λ = 320 nm). Peak 1: chlorogenic acid, peak 2: cryptochlorogenic acid. Chlorogenic acid (Sigma-Aldrich) was used for quantification of hydroxycinnamic acids. Identification of cryptochlorogenic acid was performed on the basis of UV spectra and retention times of the peaks (Chamorro et al., 2021).
